# DNA-Based Nanoswitches: Insights into Electrochemiluminescence Signal Enhancement

**DOI:** 10.1021/acs.analchem.1c01683

**Published:** 2021-07-02

**Authors:** Alessandra Zanut, Marianna Rossetti, Massimo Marcaccio, Francesco Ricci, Francesco Paolucci, Alessandro Porchetta, Giovanni Valenti

**Affiliations:** † Department of Chemistry “G. Ciamician”, University of Bologna, Via Selmi 2, 40126 Bologna, Italy; ‡ Dipartimento di Scienze e Tecnologie Chimiche, University of Rome, Tor Vergata, Via della Ricerca Scientifica, 00133 Rome, Italy

## Abstract

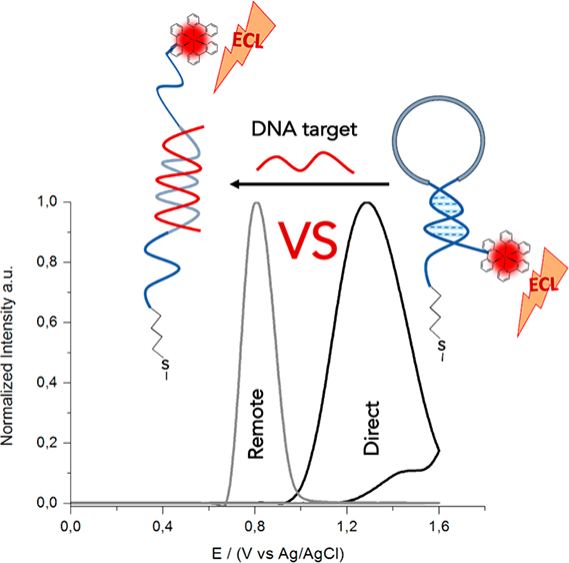

Electrochemiluminescence (ECL) is a powerful transduction technique that has rapidly gained importance as a powerful analytical technique. Since ECL is a surface-confined process, a comprehensive understanding of the generation of ECL signal at a nanometric distance from the electrode could lead to several highly promising applications. In this work, we explored the mechanism underlying ECL signal generation on the nanoscale using luminophore-reporter-modified DNA-based nanoswitches (i.e., molecular beacon) with different stem stabilities. ECL is generated according to the “oxidative-reduction” strategy using *tri-n-propylamine* (TPrA) as a coreactant and Ru(bpy)_3_^2+^ as a luminophore. Our findings suggest that by tuning the stem stability of DNA nanoswitches we can activate different ECL mechanisms (direct and remote) and, under specific conditions, a “digital-like” association curve, i.e., with an extremely steep transition after the addition of increasing concentrations of DNA target, a large signal variation, and low preliminary analytical performance (LOD 22 nM for 1GC DNA-nanoswtich and 16 nM for 5GC DNA-nanoswitch). In particular, we were able to achieve higher signal gain (i.e., 10 times) with respect to the standard “signal-off” electrochemical readout. We demonstrated the copresence of two different ECL generation mechanisms on the nanoscale that open the way for the design of customized DNA devices for highly efficient dual-signal-output ratiometric-like ECL systems.

## Introduction

Electrochemiluminescence (ECL) is a powerful analytical technique widely studied and applied from both the academic and industrial points of view.^1−4^ ECL is a luminescent phenomenon triggered by electrochemical stimulus, and thanks to the combination of electrochemical and spectroscopic methods, it shows excellent signal-to-noise ratios also in complex matrices such as cell lysates, urines, and blood. Such features established ECL as a powerful transduction method in various analytical fields such as environmental investigations, bioanalysis, and immunoassays.^5−7^ In particular, coreactant-ECL, whose generation involves tri-n-propylamine (TPrA) as the sacrificial coreactant and tris(2,2′-bipyridine)ruthenium(II) ([Ru(bpy)_3_]^2+^) as the emitting species, through an “oxidative-reduction” pathway, is among the most powerful strategies for analytical applications.^6,8^ As a surface-confined process,^6^ ECL is indeed strongly affected by the distance of the luminophores from the electrode surface,^6,9^ and a comprehensive control of spatial distribution of the ECL signal at nanometric distances is critically important in view of its applications in sensing devices. Artificially designed probes that undergo, e.g., conformational changes upon ligand binding,^10,11^ can be profitably exploited to obtain insights into the mechanisms that govern ECL signal generation at the electrode interface.

In such a context, the unique ability of DNA-based nanodevices to respond to a plethora of different biological and chemical inputs with highly reproducible nanometric conformational changes, makes them ideal candidates for investigating the generation of ECL on the nanoscale. In particular, structure-switching DNA-based probes (i.e., DNA-nanoswitches) have recently been thoroughly investigated for a number of sensing and drug-delivery applications.^12−19^ Pioneering work on DNA-based ECL sensors via an “oxidative-reduction” path using the Ru(bpy)_3_^2+^/TPrA system was done by Bard et al.^20^ In their work, they show an increased ECL when a labeled ssDNA modified with Ru(bpy)_3_^2+^ hybridizes with a complementary ssDNA immobilized on the electrode surface in a so-called “switch on” mode. This approach has opened the ECL application to DNA and RNA quantification and identification. Recently, a portable ECL-based sensor for miRNA-21 has been described which combines a switch-on ECL molecular beacon with magnetic bead-based extraction of the miRNA target sequence.^21^ Zhang et al.^22^ reported another interesting approach using a thiolated hairpin DNA assembled on gold electrodes as the recognition element and a ruthenium complex as the luminophore. However, despite the great potential of coupling DNA analysis with ECL transduction, studies on its signal generation mechanism are relatively scarce, and the signal optimization and an adequate description of the underlying mechanism still remain an open issue.

In this work, the generation of ECL from DNA-based sensors is investigated through the use of luminophore-reporter-modified stem-loop DNA probes (i.e., DNA-nanoswitch) attached to an interrogating gold electrode via self-assembled monolayer chemistry. ECL is generated according to the “oxidative-reduction” strategy using TPrA as a coreactant and Ru(bpy)_3_^2+^ as a luminophore.

Specifically, herein, we designed a couple of DNA nanoswitches (i.e., molecular beacons) that share a common recognition loop but differ in the GC base pair content of their double-stranded stem (1GC and 5GC DNA nanoswitch), resulting in a different stem stability (i.e., different free energies of their nonbinding state) and target–probe relative affinity.^11,16^ Indeed, for less stable DNA nanoswitches (i.e., 1 GC base pair in the stem, *K*_s_ > 0.1), a significant fraction of these probe is in the extended, binding-competent state even in the absence of a target. This generally produces a small signal gain of the biosensing platform upon target binding.^16^ In contrast, an overly stabilized stem (i.e., 5GC DNA-nanoswitch) reduces the observed binding affinity because it must overcome a higher free energy barrier. Consistent with this hypothesis, upon hybridization with a complementary oligonucleotide target, we observed a variation of the ECL signals, which suggests the copresence of two different mechanisms depending on the double-stranded stem stability. Moreover, we tested the performance of this sensing platform obtaining a signal-on ECL response with a signal gain 10 times higher with respect to the same system in the standard electrochemical format (i.e., amperometrically with methylene-blue-modified DNA probe). These findings could be useful hints for the design of customized DNA structures to construct highly sensitive ECL sensor platforms.

## Results and Discussion

**Figure 1:**
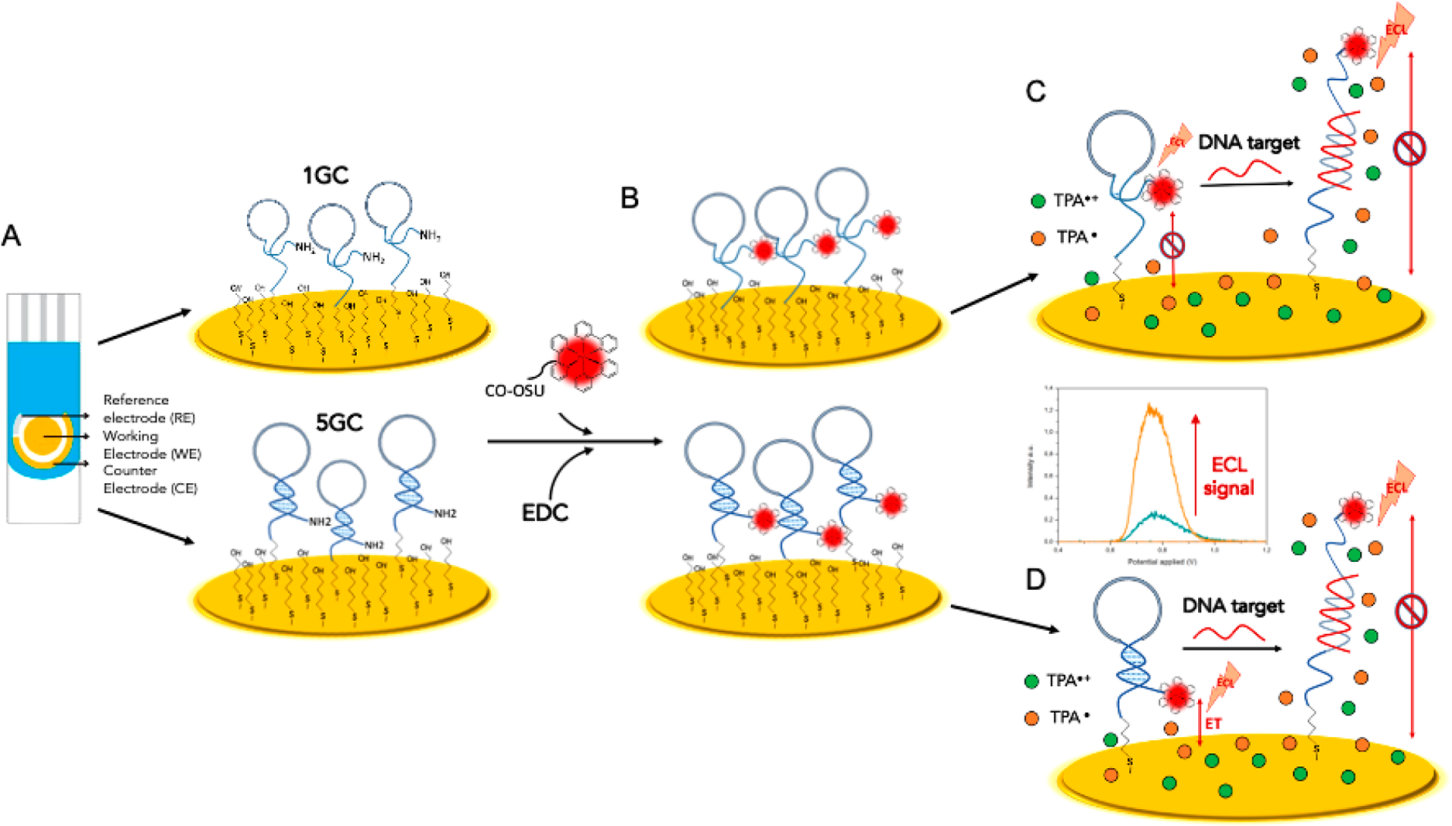
Schematic representation of the ECL-DNA sensor production. (A) A gold screen printed electrode was modified with 1GC and 5GC DNA nanoswitches with a thiol group on its 5′ end and an −NH_2_ terminal on its 3′ end, via thiol chemistry followed by a blocking in 6-mercaptohexanol. (B) 1GC and 5GC DNA nanoswitches were functionalized with ruthenium bis(2,2′-bipyridine)-(2,2′-bipyridine-4,4′-dicarboxylic acid)-*N*-hydroxysuccinimide ester (RuCO-OSU) using N-(3-(dimethylamino)propyl)-N′-ethylcarbodiimide hydrochloride (EDC) as a cross-linking agent. (C) 1GC DNA-nanoswitch ECL response before and after hybridization with the DNA target showing no direct oxidation of the luminophore at the electrode surface (Remote ECL) in both closed and open conformation. (D) 5GC DNA-nanoswitch ECL response showing direct oxidation of the luminophore (Direct ECL) in closed conformation and no direct oxidation (Remote ECL) after hybridization with the DNA target. ECL was generated using 100 mM TPrA in phosphate buffer (PB, pH = 7) as an oxidative coreactant.

The stepwise procedure to build the ECL platform is reported in detail in Figure 1: we have employed a pair of DNA nanoswitches composed of a common loop sequence (i.e., 15 nucleotides) flanked by two short self-complementary portions (i.e., stem region, six bases long) with a different content of guanosine (G) and cytosine (C) to obtain DNA nanoswitches with different stabilities (1GC and 5GC base pairing, respectively, Figure 1A). The probes were functionalized with an ECL luminophore (Figure 1B) and then hybridized with the DNA target (Figure 1C and D).

Our working hypothesis is that the conformational changes of the DNA nanoswitch can provide information on the mechanism of the ECL signal generation on the nanoscale. Specifically, our design relies on the concept that, due to the different conformations which can be populated by the DNA nanoswitch in the presence and in the absence of the DNA target counterpart, the dye (i.e., Ru(bpy)_3_^2+^) will be confined at different average distances from the electrode. Plaxco and co-workers reported that by tuning the switching equilibrium constant (*K*_s_) of stem-loop DNA probes and maintaining a constant intrinsic affinity for the target nucleic acid sequence,^23^ the signal change of the sensing platform is intrinsically controlled by the switching equilibrium constant. We estimated the free energy of the secondary structure using the nucleic acid folding predictor NUPACK, which was −2.87 kcal mol^–1^ for the 1GC DNA nanoswitch and −7.36 kcal mol^–1^ for the 5GC DNA nanoswitch, which means estimated switching constant (Ks) values of 0.1 and 5.11 × 10^–5^, respectively.

To support their incorporation into the ECL platform, each variant was provided with a thiol group on its 5′ end and an −NH_2_ terminal on its 3′ end that allow luminophore conjugation. Thiol-modified hairpin-structured DNA probes were then immobilized on the surface of a gold screen printed electrode (SPE) via S-gold chemistry and subsequently labeled with ruthenium bis(2,2′-bipyridine)-(2,2′-bipyridine-4,4′-dicarboxylic acid)-*N*-hydroxysuccinimide ester (RuCO-OSU) via forming the amide bond with the −NH_2_ moiety of DNA probes. All of the steps of the platform fabrication process were characterized by electrochemical impedance spectroscopy (Supporting Figure S1).

The ECL measurements were carried out in a phosphate buffer (PB, pH = 7), using TPrA as an “oxidative-reduction” coreactant, by scanning the potential of the electrode from 0 to 1.6 V vs Ag/AgCl.

**Figure 2:**
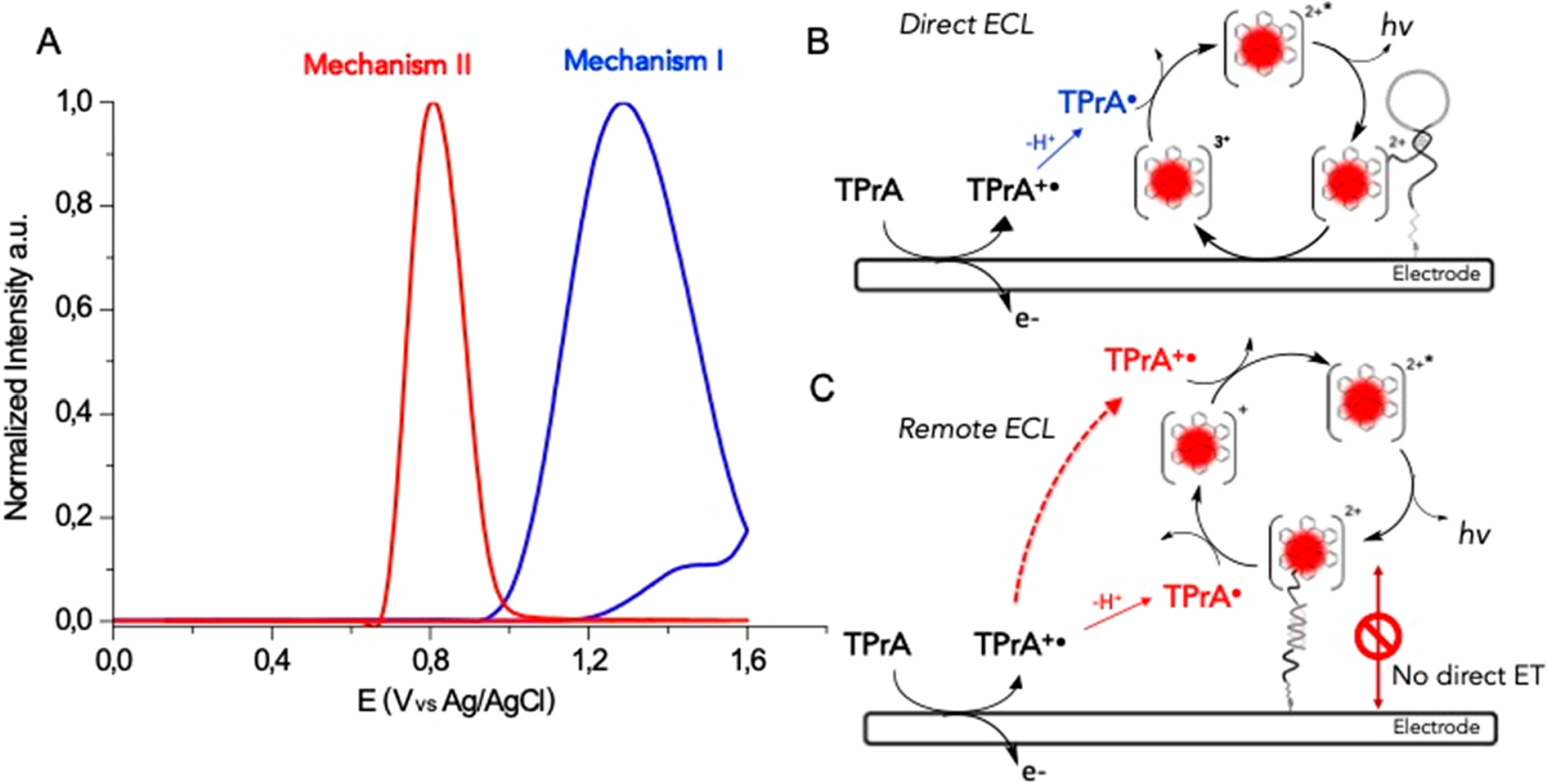
(A) Normalized ECL signals showing the two different mechanisms for the ECL generation. (B) Schematic explanation of the direct ECL mechanism present when using the 5GC DNA nanoswitch, in which [Ru(bpy)_3_]^2+^ undergoes direct oxidation on the electrode surface. (C) Schematic representation of the remote ECL mechanism in which no direct oxidation of the ECL luminophore [Ru(bpy)_3_]^2+^ occurs.

We first investigated the ECL behavior using the 5GC DNA nanoswitch in the absence (blue curve, Figure 2a) and in the presence of a DNA complementary target (i.e 30 nM, red curve in Figure 2A). As result, we observed two peaks at different potentials (i.e., 0.8 and 1.2 V) which suggest the occurrence of two different and alternative ECL generation mechanisms, i.e., mechanism I and mechanism II (Figure 2B and 2C). The observed behavior in the absence of the complementary target would suggest a mechanistic path for the generation of ECL which involves the direct oxidation of the luminophores, named *direct* ECL (mechanism I, Figure 2B), which can be described as follows:
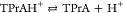
1
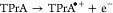
2
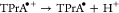
3
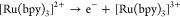
4
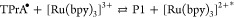
5
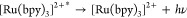
6where P1 is the degradation product of TPrA.

**Figure 3:**
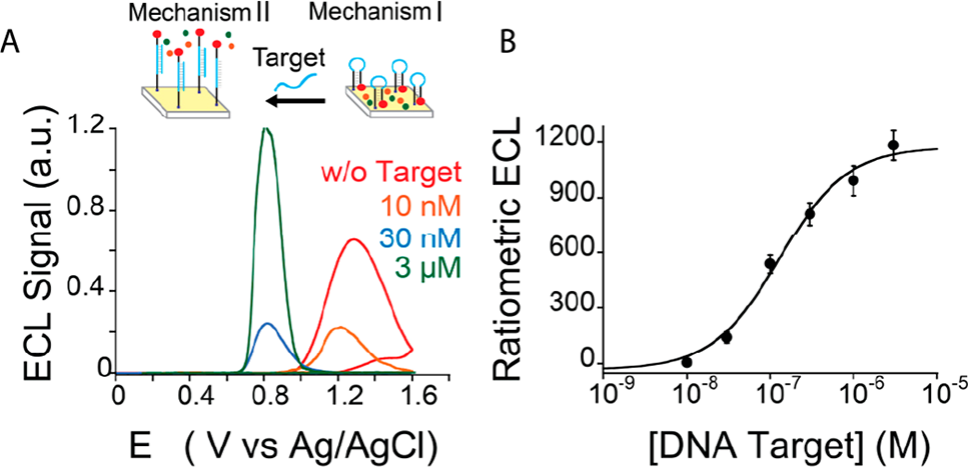
Schematic representation of the transition from mechanism I to mechanism II. (a) ECL intensity vs potential recorded using the 5GC DNA nanoswitch in the presence of a complementary DNA target (0–3 μM), obtained by applying a potential of 0–1.4 V (vs Ag/AgCl) in 100 mM TPrA in PB (pH = 7) and (b) ratiometric ECL values calculated for each DNA target concentration (see Supporting Information appendix). w/o = without.

Briefly, in this mechanism, the ECL generation is triggered by the direct oxidation of the luminophores (eq 4) and by the simultaneous oxidation of the coreactant (eq 2) followed by the deprotonation reaction that generates TPrA^●^ (eq3). TPrA^●^ and [Ru(bpy)_3_]^3+^ could react in the diffusion layer and generate the excited state (eq 5) [Ru(bpy)_3_]^2+^*. In the absence of a target, the rigid conformation of the 5GC DNA nanoswitch stem holds the lumiphore at a tunneling distance (1–2 nm) from the electrode, thus allowing its direct oxidation. Vice versa, upon hybridization with the complementary DNA target, we appreciate an ECL signal at lower potential (0.8 V), whose intensity is proportional to the target concentration (Figure 3A), suggesting a switch in the ECL generation mechanism.

This is the so-called *remote* ECL (mechanism II) that occurs when the luminophore is too distant from the electrode surface to undergo a direct electron transfer. In this case, the ECL emission is triggered exclusively by the radicals obtained by the anodic oxidation of TPrA according to a well-known mechanism originally proposed by Bard and co-workers, where the homogeneous reaction eqs 7 and 8 replace eqs 4 and 5. In this path, ECL generation requires the simultaneous presence of both radicals with sufficient fluxes to sustain the continuous generation of the excited state.
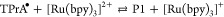
7
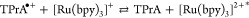
8

Mechanism II is therefore spatially limited to a region where TPrA^●+^ and TPrA^●^ concentrations are simultaneously the highest.^24,25^ Notice that the strong reductant TPrA^●^ may rapidly be scavenged at the positively biased electrode, and its steady-state concentration in proximity of the surface is therefore expectedly negligible. On the other hand, TPrA^●+^ lifetime (*t*_1/2_ ∼ 200 μs) sets a maximum distance for mechanism II, i.e., 3 μm approximately.^6^ The consistent ECL increase observed at 0.8 V for the 5GC DNA nanoswitch at higher target concentrations can then be ascribed to its switching to a target-bound stretched conformation that brings the luminophore in the region of higher TPA radicals. As said before, in the absence of the complementary target, mechanism II is instead totally inactive because of the annihilation of TPrA^●^ at the electrode.^26^

On the basis of the two limiting cases described above (Figure 2A), we developed a ratiometric-like approach for nucleic acid detection. A dual-signal-output ECL sensor presents several advantages in terms of accuracy and repeatability of the sensing system. The double signal system can effectively limit the signal interference by self-calibration of two emission signals, making the obtained results more affordable. For these reasons, ratiometric sensors are desirable for certain sensing applications aiming to monitor tight concentration intervals where systems with zero background are needed.^27,28^ For other applications such as in vivo sensing, a ratiometric approach offers the advantage that no reference signal is needed.^29^ In Figure 3, the ECL-potential profiles and corresponding ECL ratiometric responses of the 5GC DNA nanoswitch are reported. These data were obtained by adding increasing concentrations of the nucleic acid target and analyzing the response by a ratiometric method (see SI). The observed ECL response follows a “digital-like” association curve, i.e., with an extremely steep transition after the addition of increasing concentrations of the DNA target and a large signal variation. For the reasons mentioned above, in the absence of the DNA target, the ECL plot shows a well-defined peak at 1.2 V (direct Ru oxidation) while no emission is recorded in the TPrA oxidation region (0.8 V). By adding increasing concentrations of the complementary DNA strand, we observe a progressive decrease of the ECL signal at 1.2 V at 10 nM of the DNA target and the concomitant increase of the signal at 0.8 V at a DNA concentration ≥ 30 nM, the signature of the swapping from mechanism I to mechanism II.

**Figure 4:**
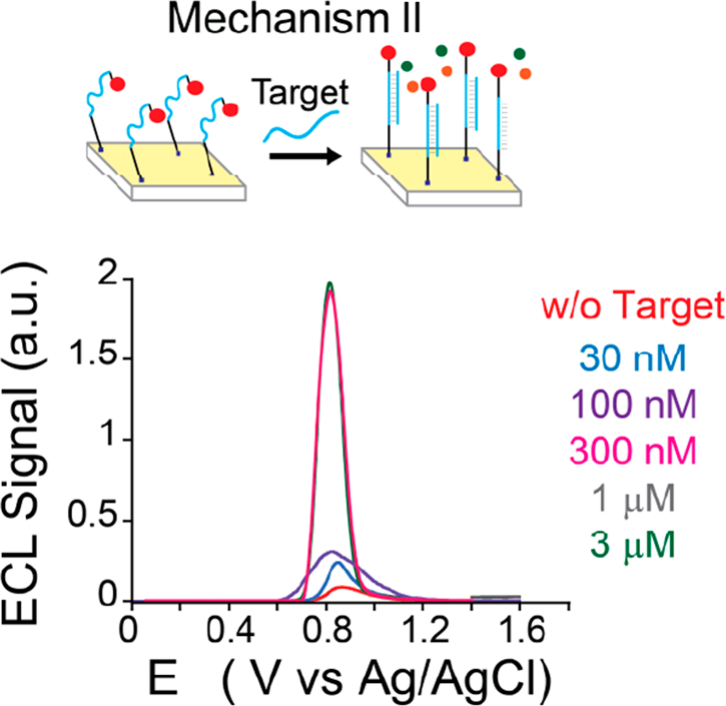
Schematic representation of mechanism II and ECL intensity vs potential recorded using a 1GC DNA nanoswitch after the addition of 0–3 μM complementary DNA target, obtained by applying a potential of 0–1.4 V (vs Ag/AgCl) in 100 mM TPrA in PB (pH = 7). w/o = without.

To further delve into this mechanism and fully exploit its potential, we studied the ECL signal transduction mediated by a stem-loop DNA probe with 1GC base pair (i.e., 1GC DNA nanoswitch) in the stem sequence. Due to the lower content of GC base pairing, the 1GC DNA nanoswitch has a lower conformational rigidity compared to the 5GC DNA nanoswitch, which may allow Ru(bpy)_3_^2+^ to move farther from the electrode surface. As a main consequence, the Ru luminophore remains, in most of the possible conformations of the DNA probe, at such a distance from the electrode surface where direct electron transfer is not permitted, thus suppressing the signal at 1.2 V. However, the greater flexibility of this probe allows the fluorophore to locate, as a function of the target concentrations, within a wider range of distances from the electrode surface than in the previous 5GC DNA nanoswitch, thus making it more fully experience the TPrA radicals gradients. In Figure 4, the ECL-potential profiles obtained with 1GC DNA nanoswitch are reported upon hybridization with various DNA target concentrations (0–3 μM). In the absence of the target, we observe an ECL signal at 0.8 V, which dramatically increases with the target concentration up to [DNA target] ∼ 100 nM where a plateau is attained, suggesting a complete saturation of the 1GC DNA nanoswitches (i.e., all in unfolded conformation). The limit of detection (LOD) for 1GC and 5GC DNA nanoswitches, taking into consideration the remote ECL signal (mechanism II), is 22 nM for the 1GC DNA nanoswtich and an LOD of 16 nM for the 5GC DNA nanoswitch (S/N = 3, Supporting Information appendix, Figure S2a and b).

To evaluate the performance of both our ECL platforms, we calculated the signal gain % (see Supporting Information) and compared them with a standard amperometric-DNA sensing platform where 1GC and 5GC methylene blue (MB)-modified DNA-nanoswitches were used (Supporting Information, Figure S3a and b).

In this case, upon hybridization with a complementary oligonucleotide target, the MB redox unit of the probe is pushed far from the electrode, thus resulting in a decrease of the electron transfer efficiency and corresponding lower Faradaic currents. It is important to point out that in amperometric DNA sensors based on molecular beacons, the maximum possible response is the lowest measurable Faradaic current, thus the signal change can be 100% at best. This is due to their intrinsic recognition mechanism which brings the redox tag far from the electrode surface in the presence of the target analyte. Remarkably, ECL sensors show instead signal gains 10 times higher (Figure S3C) when a 1GC DNA nanoswitch was used compared to a 5GC DNA nanoswitch. Moreover, by exploiting the ECL strategy, the 1GC DNA nanoswitch allowed a maximum signal gain of 2000%, which is, also in this case, 10 times higher compared to the one obtained for the 1GC DNA nanoswitch in standard amperometric-DNA operation mode.

## Conclusions

To conclude, we used luminophore-reporter-modified DNA nanoswitches, with different contents of GC bases in their stem sequence, as a model system for studying the effect of their diverse conformations on the ECL signal generation. We revealed that, by properly designing the stem nucleotide sequence, two different ECL generation mechanisms (direct for the 5GC DNA nanoswitch and remote for the 1GC DNA nanoswitch) arose from the different conformations of the DNA hairpin. DNA nanoswitches having high stem stability bring the Ru(bpy)_3_^2+^ moiety close to the electrode surface, and this results in the generation of two, previously unnoted, distinct ECL signals with the maximum at different potential values. This feature could be further exploited for developing ratiometric-like sensors where a control probe can be used to calibrate the sensing platform. Importantly, we also showed how, exploiting the remote “signal-on” mechanism, we were able to achieve a higher signal gain (i.e., 10 times) with respect to the standard “signal-off” electrochemical readout. Further studies are underway in our laboratory in order to improve the performance of this ECL platform by exploiting the reported findings coupling a coreactant with a different lifetime. Finally, the observation of two different ECL generation mechanisms dependent on the electrode/luminophore distance opens the way for the design of customized DNA devices for highly efficient dual-signal-output ratiometric ECL systems.

## Supporting Information Available

Experimental section and sensor fabrication, electrochemical characterization of the sensor, signal gain calculation and representation (PDF)
