# Supplementary material for: DNA-Based Nanoswitches: Insights into Electrochemiluminescence Signal Enhancement
Source: Anal Chem. 2021 Jul 2;93(30):10397–402. doi: 10.1021/acs.analchem.1c01683 (PMC8382220; doi:10.1021/acs.analchem.1c01683)
Supplement: Supplementary file 1 — ac1c01683_si_001.pdf [file ac1c01683_si_001.pdf]

Supporting Information

DNA-based Nanoswitches: insights into electrochemiluminescence signal enhancement.

Alessandra Zanut[a†], Marianna Rossetti[b], Massimo Marcaccio[a], Francesco Ricci [b], Francesco Paolucci[a], Alessandro Porchetta[b*], Giovanni Valenti[a*]

[a] Department of Chemistry ''G. Ciamician'', University of Bologna, Via Selmi 2, 40126 Bologna, Italy.
[b] Dipartimento di Scienze e Tecnologie Chimiche, University of Rome, Tor Vergata, Via della Ricerca Scientifica, 00133 Rome, Italy

Experimental section

Materials. All chemicals were of analytical grade and were purchased from Sigma-Aldrich (St. Louis, MO) unless otherwise indicated. Ruthenium bis(2,2'-bipyridine)-(2,2'-bipyridine-4,4'-dicarboxylic acid)-N-hydroxysuccinimide ester (RuCO-OSU) was obtained from Roche Diagnostics (Penzberg, Germany). Gold screen-printed electrodes (ref PW-AU10) were purchased from DropSens (Llanera, Spain).

High-performance liquid chromatography (HPLC)-purified DNP sequences were purchased from IBA GmBH (Göttingen, Germany). The hairpin variants were modified with a thiol-C6 group at its 5′ end and three-carbon linked to an amine group at its 3′ end. For the electrochemical detection the same hairpin sequences had a methylene blue (MB) attached by a six-carbon linker to an amine at their 3′ end.

All oligonucleotides were dissolved in buffer (200 mM Tris buffer, pH 7) at a concentration of 100 μM and then aliquot and stored at −20 °C. The DNP sequences and modification schemes of the oligonucleotide are reported here below:

1GC DNA-nanoswitch

5′ (C6-Thiol) TAT **G**TT <u>CAG GAT ATC TAG CAT</u> AA**C** ATT (C3-NH2) 3′

5GC DNA-nanoswitch

5′ (C6-Thiol) T**GC GGC** <u>CAG GAT ATC TAG CAT</u> **GCC GC**T (C3-NH2) 3′

In the above-reported sequences the underlined bases represent the stem portion. In bold are the GC bases of the loop.

DNA target

ATG CTA GAT ATC CTG

Sensor Fabrication on Screen-Printed Gold Electrodes. The gold screen-printed electrodes (Dropsense®) have a gold working electrode (4 mm diameter) and counter electrode and silver reference electrode. Electrodes were clean electrochemically through a series of cyclic voltammetry first in NaOH 0.5M scanning from 1.35 to 0.35V (500 scans, scan rate $2Vs^{-1}$), incubating for 1h in H_2SO_4 0.5M and then scanning in the same solution from 0 to 1.25V (scan rate $0.1Vs^{-1}$) until the gold peak was stable (approximately 20 scans). Then, after

carefully rinsing with deionized (DI) water, we dried them without touching the gold surface. We then deposited a 10 μL drop of the 3 μM DNA probe onto the dry electrodes and incubated for 2 h at room temperature. To prevent the evaporation of the solution, we placed the electrodes inside a Petri dish with a wet piece of paper to maintain humidity. After a final rinse with DI water (to remove the loosely adsorbed DNA), we incubated the electrodes with 2 mM 6-mercaptohexanol dissolved in Phosphate Buffer (PB, pH 7) for 1 hour at room temperature.

DNA Probe functionalization. First, Ruthenium bis(2,2'-bipyridine)-(2,2'-bipyridine-4,4'-dicarboxylic acid)-N-hydroxysuccinimide ester (RuCO-OSU) were dissolved in 100 μL di Dimethylformamide (DMF) and mixed with 150μL of buffer carbonate 0,5M ($NaHCO_3$/Na_2CO_3, pH 8). This solution was incubated for 20 minutes at room temperature. Subsequently N-(3-Dimethylaminopropyl)-N′-ethylcarbodiimide hydrochloride (EDC, 98%) was added to the solution in proportion 2:1 with respect to initial RuCO-OSU amount and 100μL of this solution were dropcasted on the electrode surface. The sensor was incubated over night at 37°C following by a five time cleaning process in Tris buffer, pH 7. The electrode was then incubated in 2 mM 6-mercaptohexanol dissolved in PB for 1 hour at room temperature and rinse carefully in PB before the hybridization with the complementary target probe.

Electrochemical and ECL Measurements. ECL and electrochemical measurements were carried out with an AUTOLAB electrochemical station (Ecochemie, Mod. PGSTAT 30).
All electrochemical measurements were performed at room temperature using an EmStatMUX potentiostat multiplexer (Palmsens Instruments, Netherland). Experimental data were collected using square wave voltammetry from 0.0 to -0.5V in increments of 0.001V *vs.* Ag/AgCl, with an amplitude of 10 mV and a frequency of 50 Hz. Peak currents were fit using the manual fit mode in the PSTrace software (of Palmsens Instrument).
For ECL measurements we exploited a home-made transparent plexiglass filled with 100 mM tri-n-propylamine (TPrA) solution in phosphate buffer (PB, pH 7) as oxidative coreactant. The sensor was connected to a specific Boxed Connector (Dropsense®) and ECL

signal was generated in cyclic voltammetry applying a potential from 0 to 1.6V and measured with a photomultiplier tube Acton PMT PD471 placed at a constant distance in front of the cell and inside a dark box. A voltage of 750 V was supplied to the PMT. The light/current/voltage curves were recorded by collecting the pre-amplified PMT output signal (by an ultralow-noise Acton research model 181) with the second input channel of the ADC module of the AUTOLAB instrument.

ECL -DNA sensor performance. The ECL platform with either 1GC or 5GC DNA-nanoswitch functionalized with RuCO-OSU was incubated with different concentration of DNA target (0-3000 nM) complementary to their loop sequence. The same procedure was done for 1GC and 5GC probe functionalized with MB.

ECL signals obtained using 5GC DNA-switch as transducer were calculated by means of a ratiometric ratio using the following formula [1]:

Ratiometric ECL = $\left(\frac{I^{II}_{ecl}}{I^{\,II}_{0ecl}} - 1 \right) / \left(1 - \frac{I^{I}_{ecl}}{I^{\,I}_{0ecl}} \right)$

Where I^{II}_{ecl} and I^{I}_{ecl} are the ECL peak intensity gained through mechanism II and I respectively; I^{II}_{0ecl} and I^{I}_{0ecl} are the initial ECL intensity for each mechanism.

ECL and EC signals were analyzed and compare by calculating the Signal Gain %, through the following formula:

% Signal Gain (ECL) = $\frac{[Iecl(T) - Iecl(0)\,]}{Iecl(0)}$ x 100

% Signal Gain (EC) = $\frac{[Iec(T) - Iec(0)\,]}{Iec(0)}$ x 100

Where $I_{ecl(T)}$ and $I_{ec(T)}$ are the ECL and EC peak intensity at a specific target concentration (0-3000nm) and $I_{ecl(0)}$ and $I_{ec(0)}$ are the ECL and EC intensity without target (close conformation).

ECL -DNA EIS characterization. EIS was performed on bare gold electrode showing a low charge electron transfer resistance that switch to an increased resistance of the double layer after the formation of the Self Assembled Monolayer (SAM) of DNA- nanoswitch. After ECL emission at 1.6 V the EIS profile went back to a low charge electron transfer resistance profile due to the formation of the oxide layer on the gold electrode which disassemble the DNA-nanoswitch SAM.

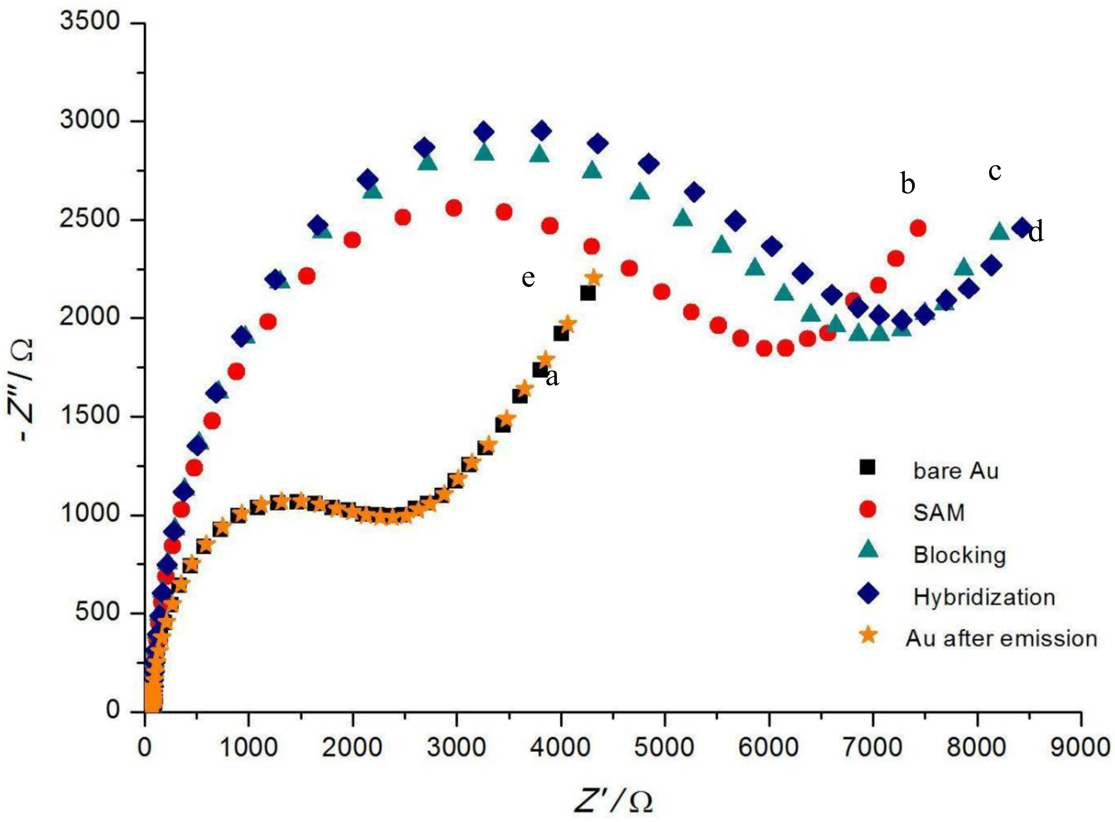

Figure S1. Nyquist plots of the electrode at different stages in 0.1M KCl and 5mM $[Fe(CN)_6]^{4-/3-}$: (a) bare Au electrode, (b) Capturing DNA/Au electrode, (c) Capturing DNA/Au electrode after blocking in 6-mercaptohexanol , (d) Capturing DNA/Au electrode after hybridization with the complementary DNA target, (e) Au electrode after ECL emission. The frequency range: 0.1–1.0Å~105 Hz. The CV scan rate: 100 mV s^{-1}, scan range: −0.2–0.6 V.

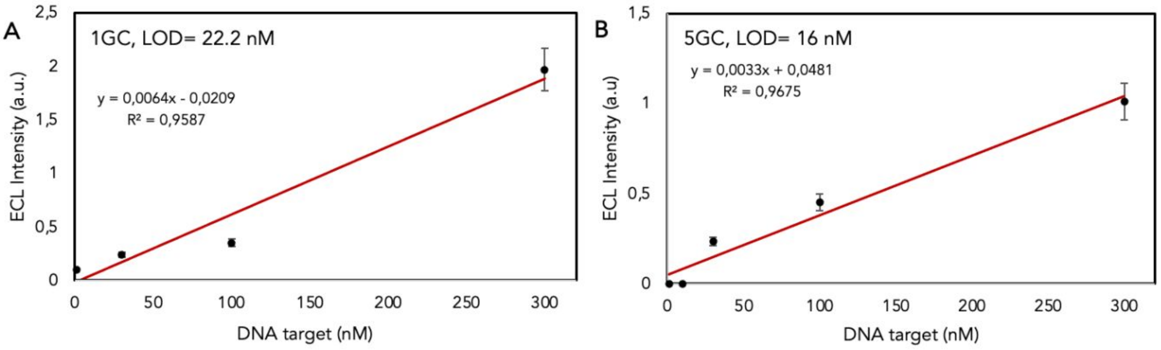

Figure S2. Remote ECL Intensity as function of the DNA target concentration for **(a)** 1 GC and **(b)** 5GC probe and respective LOD calculated by using the slope of the line obtained by fitting the linear response.

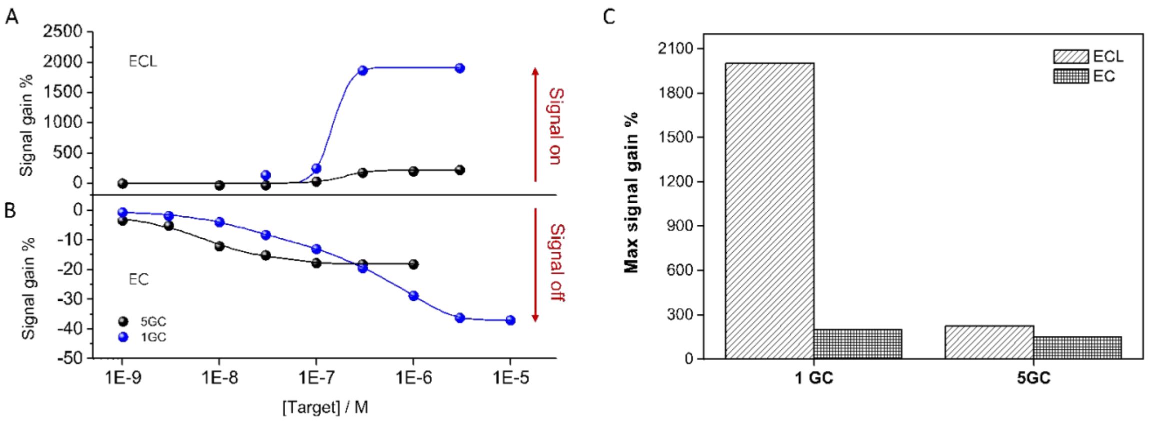

Figure S3. Signal gain % as function of the DNA target concentration for 1 GC (green) and 5GC (blue) probe calculated from **(a)** ECL signals and **(b)** electrochemical signals (EC). **(c)** Comparison between the maximum signal gain % obtained in ECL (red) and EC (blue) for the two different probes.

[1] Chen, H.; Liu, X.; Yin, C.; Li, W.; Qin, X.; Chen, C. A Dual-Signal Output Ratiometric Electrochemiluminescent Sensor for NADH Detection. Analyst 2019, 144, 5215. https://doi.org/10.1039/c9an00758j.
